# Combined treatment of PC-3 cells with ultrasound and microbubbles suppresses invasion and migration

**DOI:** 10.3892/ol.2014.2310

**Published:** 2014-07-03

**Authors:** CONG WEI, WEN-KUN BAI, YU WANG, BING HU

**Affiliations:** Department of Ultrasound in Medicine, Shanghai Jiaotong University Affiliated Sixth People’s Hospital, Shanghai Institute of Ultrasound in Medicine, Shanghai 200233, P.R. China

**Keywords:** low-frequency ultrasound, microbubble, migration, invasion, matrix metalloproteinase-2, matrix metalloproteinase-9

## Abstract

The aim of the present study was to investigate whether ultrasound treatment combined with microbubbles inhibits cell invasion and migration in androgen-independent prostate cancer (PCa) cells and to identify the probable mechanism. Ultrasound was used in continuous wave mode at a frequency of 21 kHz and with a spatial-average temporal-average intensity of 46 mW/cm^2^. Ultrasound combined with microbubbles (200 μl; SonoVue) was administered to androgen-independent human PCa PC-3 cells for 30 sec. The PC-3 cells were divided into three groups: The control group, the ultrasound group (US) and the ultrasound combined with microbubbles group (US + MB). Following treatment for 12, 24, 48 and 72 h, cell counting kit-8 was used to assess cell viability. Cell invasion and migration was measured 12 h after treatment using Transwell migration assays. Quantitative polymerase chain reaction and western blot analysis were used to evaluate the expression of the migration-associated proteins, matrix metalloproteinase (MMP)-2 and MMP-9. Cell reproduction levels in the US and US + MB groups were significantly suppressed when compared with the control group (P<0.01) following 24 h of treatment and this suppression was significantly higher in the US + MB group than in the US group (P<0.01). However, no significant differences in cell reproduction levels between the three groups were identified at 12 h (P>0.05). Ultrasound combined with microbubbles significantly suppressed the level of invasion and migration in the PC-3 cells compared with the control group (190.83±14.63 vs. 509.67±18.62, P<0.01; and 86.67±10.60 vs. 271.33±65.14; P<0.01, respectively). Furthermore, combined treatment with ultrasound and microbubbles suppressed the expression of MMP-2 and MMP-9. In conclusion, it was found that ultrasound combined with microbubbles suppressed invasion and migration in human PCa PC-3 cells via downregulation of MMP-2 and MMP-9.

## Introduction

Prostate cancer (PCa) is the second most commonly diagnosed cancer. Accounting for 14% (903,500) of the total number of new cancer cases and 6% (258,400) of the total number of cancer-related mortalities in males in 2008, PCa is the sixth leading cause of cancer-related mortality in males (1). The growth and maintenance of cancerous cells in the early stages of PCa is androgen-dependent, and therapy often involves androgen deprivation (2). In the later stages, therapeutic alternatives are not available, as the tumor becomes androgen-independent. There is an association between our poor understanding of the molecular mechanisms that underlie disease progression with regard to invasion and metastasis, and the lack of successful therapies for advanced PCa (3). PCa has a significant impact on survival and patient quality of life due to its high potential for metastasis to other areas of the body (4). Clinical observations have shown that approximately one-third of PCas invade the surrounding tissues, metastasize to distant organs and cause subsequent mortality, despite current therapies. The survival of a patient with PCa is directly associated with the spread of the tumor (5).

As ultrasound is non-invasive and perceived as safe, with relatively low costs, the technique is widely used for the imaging of soft tissues. Ultrasound can also be used therapeutically; specifically, the technique has been investigated in certain preclinical therapeutic studies and shown to mediate apoptosis in numerous *in vitro* and *in vivo* experimental systems (6–8). Furthermore, the greater susceptibility of cancer cells to ultrasound therapy has been demonstrated in comparison to normal cells (9,10), resulting in its eventual application in the treatment of cancer. In addition, enhancement of the apoptotic effect that ultrasound induces in tumors may occur through the use of porphyrins, anticancer drugs and microbubbles (10,11). Ultrasound combined with microbubbles exhibits the ability to induce tumor apoptosis, however, it remains unclear whether the technique can inhibit cell invasion and metastasis.

In cancer cells, the matrix metalloproteinases (MMPs) are overexpressed and are involved in the invasion and metastasis of various cancer cells (12). MMP-2 and -9 are components of the basement membrane and thus, their involvement is indicated in the invasion and metastasis of malignant cancers. Therefore, the inhibition of MMP activity is significant for the prevention of cancer, particularly the cancer promotion process (13).

In the present study, an ultrasound frequency of 21 kHz was used, with a spatial-average temporal-average intensity (ISATA) of 46 mW/cm^2^. Human PCa PC-3 cells were treated with low-frequency and low-energy ultrasound combined with microbubbles. Following treatment, the antimetastatic effect of the ultrasound combined with microbubbles was determined in the PC-3 cells using migration and invasion assays. Additionally, changes in the MMP-2 and MMP-9 mRNA and protein levels were evaluated.

## Materials and methods

### Cell culture

The androgen-independent human PCa PC-3 cell line was obtained from the Cell Bank of the Chinese Academy of Sciences (Shanghai, China). The cells were grown in Dulbecco’s modified Eagle’s medium (DMEM; Gibco-BRL, Grand Island, NY, USA) supplemented with 10% heat-inactivated fetal bovine serum (Invitrogen Life Technologies, Carlsbad, CA, USA) at 37°C in a humidified atmosphere containing 5% CO_2_. The PC-3 cells were resuspended at a density of 1×10^6^ cells/ml and placed into 1.5-ml polystyrene test tubes. Each tube contained a 1-ml suspension of PC-3 cells. The tubes were 13 mm in diameter, with planar bottoms, allowing them to be positioned closer to the ultrasound probe.

### Ultrasound apparatus and microbubbles

Ultrasound treatment was performed using a FS-450 ultrasonic processor (Shanghai Institute of Ultrasound in Medicine, Shanghai, China) in combination with the SonoVue microbubble echo-contrast agent (Bracco Imaging SpA, Milan, Italy). The FS-450 ultrasonic processor was equipped with a built-in digital timer and an intensity regulator. The probe frequency was fixed at 21 kHz, with an intensity of 46 mW/cm^2^. In all experiments, ultrasound was generated by a 21-kHz ultrasound probe using the continuous wave mode. The duration of treatment was 30 sec. The ultrasound probe was cylindrical with a diameter of 13 mm, identical to that of the test tubes. The experimental setup for ultrasound exposure has been shown in our previous study (14).

The SonoVue agent used was a lipid-shelled ultrasound contrast agent composed of microbubbles filled with sulfur hexafluoride gas. The microbubbles were 2.5–6.0 μm in diameter. Prior to use, the SonoVue agent was reconstituted in 5 ml of phosphate-buffered saline at a concentration of 2–5×10^8^ microbubbles/ml.

In all experiments, the cells were divided into three groups: The control group (no treatment); the ultrasound group (US); and the ultrasound combined with microbubbles group (US + MB; 200 μl SonoVue). Each group contained three samples.

### Measurement of cell proliferation

Following treatment, each group of cells was seeded at a density of 3×10^3^ cells/well in 96-well plates. Following incubation for 12, 24, 48, or 72 h, 100 μl cell counting kit-8 (Dojindo Laboratories, Kumamoto, Japan) was added. The plates were then incubated for an additional 3 h. The optical density of each well was measured using a microculture plate reader (Bio-Tek Instruments, Inc., Winooski, VT, USA) at a wavelength of 450 nm (15).

### In vitro migration and invasion assays

For the Transwell migration assays, following treatment, 5×10^4^ PC-3 cells were plated in the top chamber with the non-coated membrane (24-well insert; pore size, 8 μm; Corning Inc., Lowell, MA, USA). For the invasion assays, 5×10^4^ cells were plated in the top chamber with a membrane (24 well insert; pore size, 8 μm; Corning Inc.) coated in Matrigel (1 mg/ml; BD Biosciences, San Jose, CA, USA). In the two assays, the cells were plated in serum-free DMEM medium, and medium containing 10% serum was used as a chemoattractant in the lower chamber. The cells were incubated for 12 h at 37°C in an atmosphere of 5% CO_2_. Following incubation, a cotton swab was used to remove the non-migrated cells in the upper chamber and the filters were individually stained with 2% crystal violet (Beyotime Institute of Biotechnology, Nantong, China). The migrated cells adhering to the underside of the filter were examined, counted and images were captured under a light microscope (magnification, ×200; Olympus IX70; Olympus Corporation, Osaka, Japan) (16).

### Quantitative polymerase chain reaction (qPCR)

To quantitatively determine the mRNA expression level qPCR was performed. The total RNA of each clone was extracted using TRIzol (Invitrogen Life Technologies) according to the manufacturer’s instructions. Reverse-transcription was carried out using M-MLV, and cDNA amplification was performed using the SYBR Green Master Mix kit (Invitrogen Life Technologies) according to the manufacturer’s instructions. MMP-2 and MMP-9 genes were amplified using specific oligonucleotide primers, and the human glyceraldehyde-3-phosphate dehydrogenase (GAPDH) gene was used as an endogenous control. The PCR primer sequences used were as follows: MMP-2 forward, 5′-TTGGTGGGAACTCAGAAG-3′ and reverse, 5′-TTGCGGTCATCATCGTAG-3′; MMP-9 forward, 5′-GTGGCACCACCACAACATCAC-3′ and reverse, 5′-CGCGACACCAAACTGGATGAC-3′; and GAPDH forward, 5′-CAACGAATTTGGCTACAGCA-3′ and reverse, 5′-AGGGGTCTACATGGCAACTG-3′. Data were analyzed using the comparative Ct method (2^−ΔΔCt^) (17). Three separate experiments were performed for each clone.

### Western blot analysis

Subsequent to 24 h, the treated and untreated cells were harvested and lysed, and the supernatants were separated from the cell debris by centrifugation at 13,000 × g for 15 min at 4°C. Aliquots containing 30 μg of total protein were separated by SDS-PAGE and transferred onto nitrocellulose membranes. The membranes were then probed with primary rabbit monoclonal antibodies against MMP-2 and -9 (Santa Cruz Biotechnology, Inc., Santa Cruz, CA, USA) at 4°C overnight. The membranes were subsequently probed with a goat anti-rabbit secondary antibody conjugated with horseradish peroxidase (Santa Cruz Biotechnology, Inc.) and visualized by electrochemiluminescence. Protein band densities were quantified using Bio-Rad Quantity One software (Bio-Rad, Hercules, CA, USA).

### Statistical analysis

Data are expressed as the mean ± standard deviation. Different groups were compared using a paired t-test and P<0.05 was considered to indicate a statistically significant difference.

### Approval

This study was approved by the Ethics Committee of Shanghai Jiao Tong University Affiliated Sixth People’s Hospital (Shanghai, China).

## Results

### Measurement of cell proliferation

**Figure 1:**
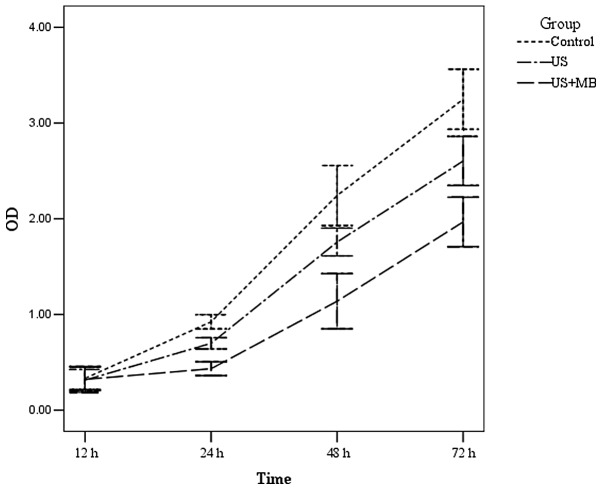
Cell proliferation in the control, US and US + MB groups at 12, 24, 48 and 72 h after treatment. OD, optical density; US, ultrasound group; US + MB, ultrasound in combination with microbubbles group.

Cell reproduction levels in the US and US + MB groups were significantly suppressed when compared with the control group (P<0.01) following treatment for 24 h and this suppression was significantly higher in the US + MB group compared with the US group (P<0.01). However, no significant difference in cell reproduction levels was identified between the three groups following 12 h of treatment (P>0.05) (Fig. 1).

### In vitro migration and invasion assays

**Figure 2:**
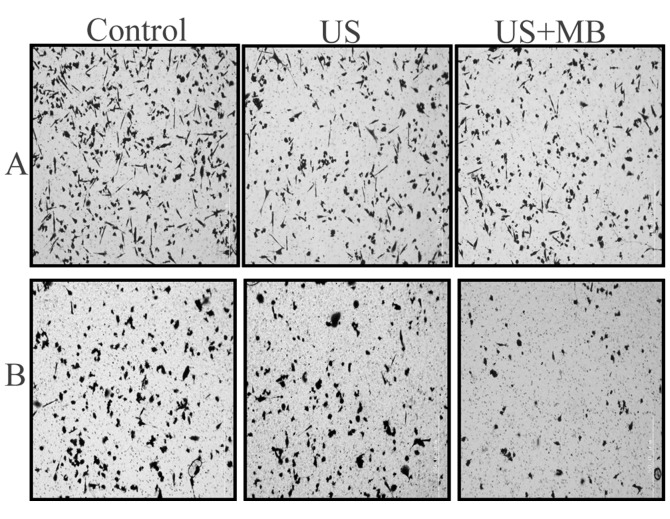
Ultrasound treatment combined with microbubbles suppresses the (A) migration and (B) invasion of PC-3 cells. US, ultrasound group; US + MB, ultrasound in combination with microbubbles group (magnification, ×200).

To evaluate the migration potential of the PC-3 cells using ultrasound combined with microbubbles *in vitro*, migration assays were performed. The result revealed that PC-3 cell motility was suppressed in the US and US + MB groups compared with the control group (Fig. 2A; Table I; P<0.01). However, this suppression was significantly higher in the US + MB group than in the US group (P<0.01). To evaluate the effect of ultrasound combined with microbubbles on the invasive ability of the PC-3 cells, an *in vitro* invasion assay was performed. The number of cells passing through the filter was markedly less in the US and US +MB groups compared with the control group (Fig. 2B; Table I; P<0.01). Furthermore, this suppression was significantly higher in the US+MB group than in the US group (P<0.01). These results indicated that ultrasound combined with microbubbles suppresses PC-3 cell migration and invasion *in vitro*.

### MMP-2 and MMP-9 expression following treatment

**Figure 3:**
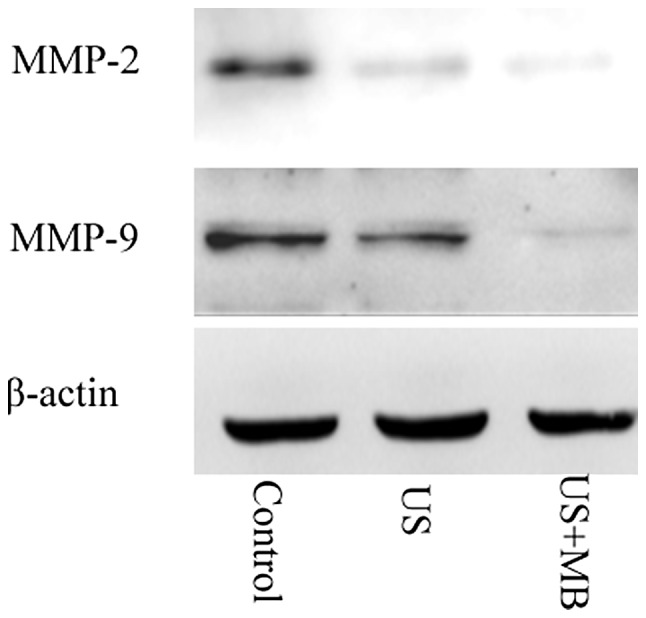
Expression of MMP-2 and MMP-9 protein following treatment. MMP, matrix metalloproteinase; US, ultrasound group; US + MB, ultrasound in combination with microbubbles group.

The expression of MMP-2 and MMP-9 mRNA and protein was investigated following treatment. It was found that the MMP-2 and MMP-9 levels in the US and US + MB groups were significantly suppressed compared with the control group (P<0.01), and that this suppression was significantly greater in the US + MB group than in the US group (P<0.01). (Tables II and III; Fig. 3).

## Discussion

The biophysical modes of ultrasound exhibit three types of effect: Thermal, cavitational and mechanical (18). Low-frequency ultrasound induces predominantly mechanical and cavitational effects, as the thermal effect results in only a neglible temperature increase (19). In the present study, regular cell media that had not been degassed or air-saturated was used to avoid any resulting cell changes. Thus, the cell media may have contained air bubbles that were able to produce cavitation (20). Following ultrasound exposure, insonation may be used to intentionally collapse microbubbles suspended in liquid, resulting in the production of a mechanical force on the cellular membrane, and the destruction of adjoining cellular membrane integrity. The cavitation effect is more intense following the addition of microbubbles to the cell suspension compared with administration of ultrasound alone. Collapsing microbubbles and the resultant cavitation bubbles create impulsive pressures, including liquid jets and shock waves, which result in cell apoptosis, enzyme inactivation and the denaturation of proteins. Neighboring cells are also affected by these pressures, as the propagation distance of the shock-wave from the center of a cavitation bubble with the potential to damage a cell membrane is greater than the maximum radius of the cavitation bubble (21).

Clinically, ultrasound is of low intensity at a value of 0–0.5 W/cm^2^, of medium intensity at a value of 0.5–3 and of high intensity at a value of >3 W/cm^2^ (22). As high energies are involved in ultrasound treatment, cell lysis forms the predominant effect, which possibly masks additional effects on surviving cells (23,24).

In the present study, cell proliferation was measured at 12, 24, 48 and 72 h post-ultrasound treatment. The results indicated that ultrasound combined with microbubbles suppressed the proliferation of the PC-3 cells following 24 h of treatment. In our previous study, an ultrasound frequency of 21 kHz, with an ISATA of 46 mW/cm^2^ and a continuous wave mode was used to treat PC-3 cells for 30 sec. It was found that ultrasound combined with microbubbles was able to induce apoptosis in PC-3 cells (25). These results indicated that ultrasound combined with microbubbles inhibits cell proliferation via a mechanism involving apoptotic induction. In our previous study, cell viability was evaluated immediately following treatment. The results indicated that ultrasound alone and ultrasound combined with microbubbles exhibited minimal effects on the viability of the PC-3 cells and cause minimal induction of cell lysis (25).

One of the key stages in cancer invasion and metastasis is the degradation of the extracellular matrix. Notably, MMP-2 and MMP-9 have been demonstrated to be significant in this process (26). Numerous studies have shown that the inhibition of MMP expression and/or the inhibition of the activities of MMP enzymes may be used as early targets for preventing cancer metastasis (27–29). In addition, i*n vitro* studies have revealed that the expression of MMP-2 and MMP-9 is associated with the high invasive and metastatic potential of PCa cell lines (30). The results from the migration and invasion assays of the current study also demonstrated that ultrasound combined with microbubbles inhibited the invasion and migration of PC-3 cells *in vitro*. In addition, the results revealed that the antimetastastic effects of ultrasound combined with microbubbles were associated with the inhibition of enzymatically degradative metastatic processes in the PC-3 cells. Ultrasound combined with microbubbles inhibited the activities of MMP-2 and -9, which are involved in the degradation of the extracellular matrix and thus, are important in cancer cell migration and invasion.

Collectively, ultrasound treatment combined with microbubbles exhibits antimetastatic activity and thus has the potential to be developed into an antimetastatic agent for PCa. The possible signal pathways targeted by ultrasound combined with microbubble treatment may inhibit migration and invasion in PC-3 cells via the downregulation MMP-2 and -9. Therefore, ultrasound treatment combined with microbubbles presents considerable promise as an antimetastatic treatment for androgen-independent PCa.

## Figures and Tables

**Table I:** Number of migrating and invasive cells following treatment.

Group	Migration	Invasion
Control	509.67±18.62	271.33±65.14
US	386.67±44.23^a^	180.67±13.29^a^
US + MB	190.83±14.63^a^,^b^	86.67±10.60^a^,^b^

a P<0.01 vs control.

b P<0.01 vs US.

US, ultrasound group; US + MB, ultrasound in combination with microbubbles group.

**Table II:** Expression of MMP-2 and MMP-9 mRNA following treatment.

Group	MMP-2 mRNA	MMP-9 mRNA
Control	11.64±1.02	12.69±1.80
US	5.65±1.17^a^	3.05±0.49^a^
US + MB	1.47±0.51^a^,^b^	0.15±0.07^a^,^b^

a P<0.01 vs control.

b P<0.01 vs US.

MMP, matrix metalloproteinase; US, ultrasound group; US + MB, ultrasound in combinatin with microbubbles group.

**Table III:** Expression of MMP-2 and MMP-9 protein following treatment.

Group	MMP-2	MMP-9
Control	0.80±0.06	0.73±0.08
US	0.55±0.09^a^	0.47±0.08^a^
US + MB	0.25±0.05^a^,^b^	0.15±0.05^a^,^b^

a P<0.01 vs control.

b P<0.01 vs US.

MMP, matrix metalloproteinase; US, ultrasound group; US + MB, ultrasound in combination with microbubbles group.
